# The state of online citizen science in Mongolia and its potential for environmental challenges

**DOI:** 10.1371/journal.pone.0289924

**Published:** 2023-08-14

**Authors:** Anudari Batsaikhan, Stephan Jung, Stephan Hachinger

**Affiliations:** 1 Leibniz Supercomputing Centre (LRZ) of the Bavarian Academy of Science and Humanities, Garching bei München, Germany; 2 Rachel Carson Center for Environment and Society, Munich, Germany; 3 TUM School of Life Sciences, Ecoclimatology, Technical University of Munich, Freising, Germany; The City College of New York, UNITED STATES

## Abstract

Mongolia is a sparsely populated Asian country covered by vast steppes, deserts, and forests. Few studies have been conducted on Online Citizen Science (OCS) activities in Mongolia. This study aims to analyze the state of OCS in Mongolia and, in a further step, to place it in an international context by comparing it with Germany and Japan, where OCS is already well established. Mongolia faces several environmental challenges, including climate change, land-use change, and intensive urbanization. OCS can help address these environmental challenges. Quantitative, qualitative, and literature-based analyses were conducted in this study. OCS has become more relevant in Mongolia since 2013, where projects have been introduced internationally rather than locally. A comparison with Germany and Japan showed that the use of web technologies and the degree of citizen participation in OCS projects are similar in these countries; however, the link to the United Nations Sustainable Development Goals (UN SDGs) may differ. To better respond to citizen needs and environmental challenges, additional local projects must be developed. Mongolia has the potential to enhance environmental monitoring and the networking of various actors using web technologies in citizen science.

## Introduction

Citizen science attempts to increase citizen involvement in scientific research to generate new knowledge [1, 2]. OCS incorporates information and communication technologies (ICTs), especially web-based and mobile technologies, to collect, analyze, and display data on a particular topic [3, 4].

Incorporating ICTs offers the advantages that citizens can participate regardless of their location, that learning outcomes can be improved through interactive interfaces, and that ICT skills can be enhanced through interactions with web / mobile platforms and digital devices [3, 5]. ICTs have shown their importance in citizen science, especially in disaster management (e.g., in response to earthquakes and COVID-19) [6, 7].

### Related work

Existing studies on OCS cover the drivers of participation [6, 7], effects on learning and scientific literacy [3, 5], and drivers of data quality and quantity [8]. Furthermore, there have been studies on conceptual frameworks and design considerations for developing online projects [9, 10], recommendations for developing mobile citizen science applications [11], and case studies on individual projects [12, 13]. The parameters considered in the aforementioned studies are mostly of an applied nature and are related to various aspects of the citizen science process.

Regarding the state of citizen science, existing studies used different approaches to analyze the dynamics in citizen science at the global level. In one approach, spatiotemporal patterns based on observations on the Zooniverse platform were investigated and quantified [14]. In another study, it was quantified, based on citizen science publications, where citizen science projects are conducted and which was then visualized on a global map [15].

A qualitative survey was conducted for Europe that examined citizen science projects in terms of their type, perceived impact, added value, and challenges, among others [16]. In a study conducted for Africa, citizen science projects were first identified through web-based research, and in a subsequent step, surveys were used to quantify the use of web technologies, educational tools among others [17].

At the country level, web-information based studies on citizen science projects, for example for Brazil [18], Germany [19], Japan [20], and South Africa [21] examined starting year, target age group, research field, main activities, the level of citizen’s participation (Haklay’s classification), web technologies, the linkage to UN SDGs, number of participants, number of observations, the type of the involved institutions, among others. Survey based studies, for example for Ireland [22] and Spain [23] examined awareness of citizen science in the educational institutions, the citizen science project’s outputs, the level of citizen’s participation (Haklay’s classification), training of participants, among others.

There is no unified approach for analyzing the state of citizen science in a particular country, thus the investigated parameters highly depend on the respective study.

The aim of this study was to analyze the state of OCS in Mongolia. It focused on frequently used parameters (see above) with special focus on OCS. The following section briefly describes Mongolia.

### Citizen science in Mongolia

Mongolia is a country in Asia, located between Russia, to the north, and China, to the south. It has an area of 1, 564, 116 km^2^, a population of approximately 3 million, and is one of the most sparsely populated countries in the world [24]. Mongolia’s largest city is the capital city, Ulaanbaatar, with an area of 4,704 km², and is inhabited by approximately half the country’s population [25]. The country is covered by steppes, with mountains to the north and west and the Gobi Desert to the south. It is characterized by a cold, dry, and continental climate. Its climate has significantly limited crop production, and the agricultural sector has traditionally been driven by nomadic livestock farming.

**Fig 1:**
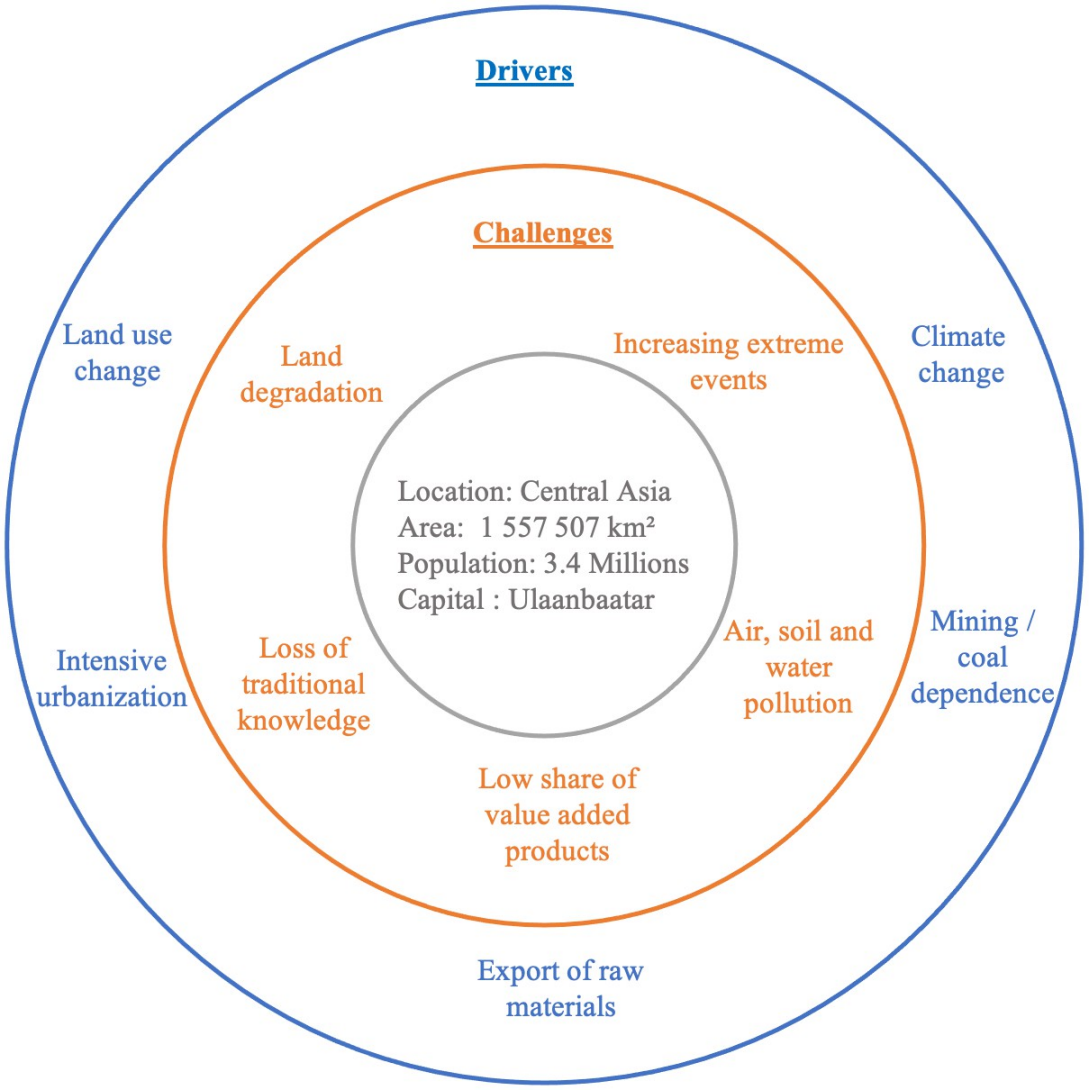
Overview of some of the main environmental challenges (orange) and their underlying drivers (blue) in Mongolia.

Mongolia has faced social and environmental challenges over the last three decades (Fig 1) that have affected its ecosystems and livelihood. Climate change in Mongolia is characterized by an increase in extreme events, such as summer droughts and heavy snowfall in winter [26]. Human activities, such as the large-scale mining of coal, copper, and gold, have had various impacts on water resources, soil, and vegetation [27, 28]. There is a high dependence on the export of raw materials; the manufacturing and export of value-added products are rather low. Due to Mongolia’s limited production of goods, various common products are imported from other countries [29, 30]. Influenced by various factors, such as climate change and urbanization, traditional nomadic livestock farming practices are changing, which consequently affect ecosystems and biodiversity [31]. There is a strong trend of migration from rural areas to large cities, especially Ulaanbaatar. Enhanced population growth in cities has exacerbated serious problems related to exhaust gases from road traffic and the use of unsustainable heating systems [32].

Citizen science is still relatively new in Mongolia and has recently increased, in part due to the activities of nonprofit organizations (NPOs), such as Public Lab Mongolia founded in 2018 [33]. To date, there is scarce literature on the development and state of citizen science in Mongolia. Between 2011 and 2013, greater publicity was mainly gained through the OCS project (“Field Expedition Mongolia”), which was co-organized by international researchers supported by National Geographic [34]. Faced with environmental challenges, citizen science research in Mongolia can form an important initiative to strengthen environmental monitoring, and thus help preserve cultural heritage and biodiversity. Additionally, citizen science research can strengthen local communities and promote public engagement in science. ICT-based OCS can overcome traditional data-collection methods. Thus, Mongolia is an excellent entry point for citizen science. Unlike many other countries, OCS activities in Mongolia have not yet been thoroughly researched.

This study examined the state of OCS in Mongolia and its potential using quantitative, qualitative, and literature-based analyses. The available literature-based information on OCS related to Mongolia, the number of OCS projects in Mongolia, and the OCS observations and observers from Mongolia were quantified. Quantification was based on the Google Scholar search engine and iNaturalist, a global citizen science web platform.

The qualitative analysis addresses the use of web technologies, link to the UN SDGs, and the degree of citizen participation in OCS projects in Mongolia. The UN SDGs are 17 global goals adopted by all UN member states to achieve a more sustainable future [35]. These goals can serve as blueprints that citizen science projects can use as guidance to contribute to sustainable development. Due to Mongolia’s multifaceted challenges, they were included in the analysis.

The state of OCS in Mongolia was then compared with that in Germany and Japan. This study considers both countries, each with different characteristics, as frontline cases for OSC in Mongolia. A country like Mongolia, where citizen science is still in its early stages, can learn from these countries, which is the primary reason for selecting Japan and Germany for comparison. Germany has one of the most well-established citizen science policies in Europe and is one of the limited number of countries with a national central platform for citizen science [36, 37]. Japan is known globally for its successful implementation of citizen science for disaster management [38, 39], from which approaches for dealing with environmental challenges can be derived. A summary of the environmental challenges in Mongolia and how OCS can contribute to coping with these issues is provided in this study. Already established practices from other countries that could enhance OCS advantages for Mongolia are highlighted.

## Materials and methods

### Quantitative analysis

#### Keyword search

**Fig 2:**
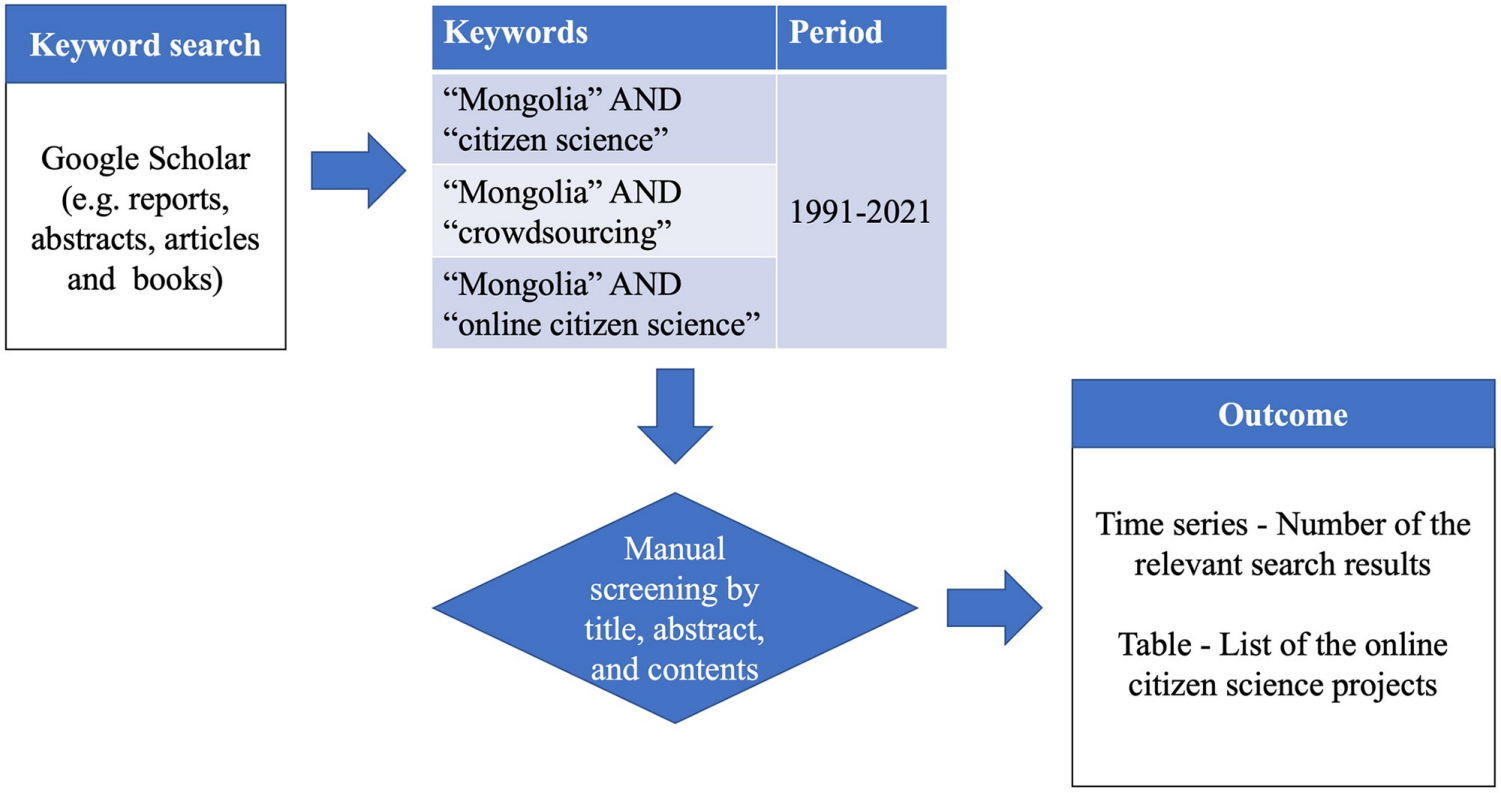
Workflow for the keyword search and expected outcome.

In this study, a keyword search method was used to analyze the current state of OCS in Mongolia (Fig 2). Previous studies have shown that keyword searches are an effective means of gathering information on topics of interest [40–45]. This study used Google Scholar, which includes not only peer-reviewed articles, but also abstracts, reports, research articles, and books [46], to analyze OCS in Mongolia based on the amount of information found through keyword searches.

For the keyword search, "citizen science," "online citizen science," and the most commonly used synonym "crowdsourcing" were used. All search terms were entered together with "Mongolia.” The word "crowdsourcing" is especially viewed as a synonym for "OCS," which has been frequently used since the 2000s and generally refers to online activities that people collaborate on [47, 48]. The search concentrated on English-language results.

Second, all search results were manually screened to determine whether they were related to citizen science in Mongolia. Relevant search results included all hits concerning projects, movements, and activities.

Finally, the search results from the second step were used to identify individual OCS projects. Only projects showing a clear link to citizen science were included. This list could also include projects that are no longer active. There may be other citizen science projects in Mongolia that have been reported after the period included in this study (1991–2021), or they may not include any of the listed search terms in their publications; therefore, they may not be identified within this study.

#### Platform search

Several globally operating citizen science platforms are oriented towards project organization or user participation. Previously, the analysis of such platforms provides valuable results on activities related to OCS [45, 49–51]. The key OCS platforms [52] are SciStarter [53] and Citsci [54], which function as mutual learning platforms and provide useful guidelines and tools for OCS [55, 56], whereas iNaturalist [57] and Zooniverse [58] enable platform users worldwide to find, interact, and join biodiversity-related citizen science projects [52, 59]. The SciStarter, Citsci, and Zooniverse platforms did not show any content with a focus on Mongolia. The relevant content on Mongolia was found only on the iNaturalist platform. As a result, the iNatualist platform [57] was used to explore the presence of citizen science observations from Mongolia on a global citizen science web platform iNaturalist enables worldwide participation in citizen science by following a large common interest, in this case, biodiversity monitoring.

In the analysis of iNaturalist, the number of observations and observers in Mongolia were determined using the R software in combination with the "rinat" [60] package for each year from 2008 (the initial year of iNaturalist) to 2021. Of the 13 iNaturalist categories, the four categories with the highest number of observations were identified.

### Qualitative analysis

A qualitative analysis was performed based on individual projects identified in the keyword search. All data were manually extracted from the project’s website/web app and social media. The qualitative analysis consisted of the following aspects.

#### Use of web technologies

Based on the methodology used by Batsaikhan et al. (2022), the use of web technologies in individual OCS projects was examined in the following categories: online platforms, educational tools, social media, and data sharing.

#### Link to UN SDGs

It was examined which UN SDGs that individual projects could be linked to based on project descriptions. The screening process included project descriptions and objectives.

#### Degree of citizen participation

The extent to which citizens are involved in OCS projects was analyzed. For this purpose, the existing classification method by Haklay et al. (2013) was applied, in which citizen science projects are classified into four levels depending on the degree of citizen participation [61]. In Level 1, "Crowdsourcing," citizen involvement was relatively low, with citizens participating as sensors, primarily collecting data for the project without prior training. In Level 2 "Distributed Intelligence," citizens acted as basic interpreters by collecting, processing, and interpreting data. In this case, prior training was required. Level 3, "participatory science," involved citizens in problem definition and data collection. Most citizen science projects belong to this class. Level 4, "Extreme," had the highest level of citizen participation, as citizens were involved in the project at various stages, from problem definition to data collection and analysis. To assign one of the four levels to the projects, the project platforms were manually screened for possible participation. Levels was assigned to each project based on the participation type.

### Literature-based analysis

The results for the state of OCS in Mongolia were placed in an international context by comparing them with Japan and Germany. A literature review was conducted to provide a brief overview of citizen science in Germany and Japan. The literature review concentrated on the aspects that were analyzed for Mongolia: the use of web technologies [20, 62], link to UN SDGs [63], and Haklay’s classification [20, 63].

A literature review was conducted to address the approaches to OCS that could potentially contribute to coping with environmental challenges, the output of which is included in the discussion.

## Results

### Quantitative analysis

#### Keyword search

**Fig 3:**
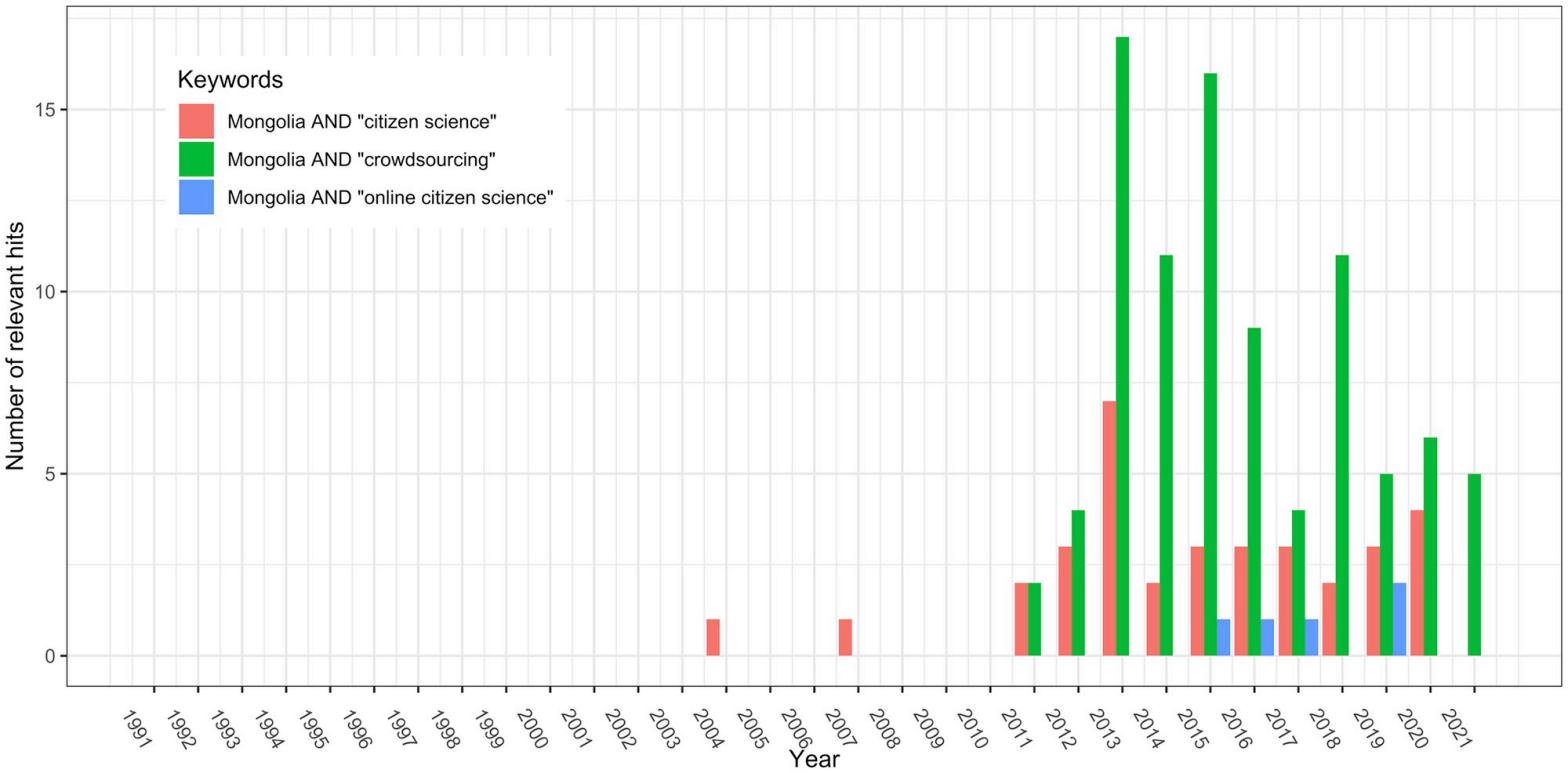
Time-series of the number of search results relevant to OCS in Mongolia from 1991–2021. The red bar shows results corresponding to "Mongolia" and "citizen science," the green bar represents "Mongolia" and "crowdsourcing," and the blue bar indicates "Mongolia" and "online citizen science".

From 1991 to 2021, the search returned 34 relevant hits (out of 1,111 total hits) after manual screening for the terms "Mongolia" and "citizen science"; 89 relevant hits (out of 1,107 total hits) for the terms "Mongolia" and "crowdsourcing"; and four relevant hits (out of 9 total hits) for "Mongolia" and "online citizen science.” When several search terms appeared in a document, the search results may have overlapped. The initial search results were obtained in 2004. A significant increase was observed in 2013 (Fig 3).

Using the search terms "Mongolia" and "citizen science,” seven projects were identified, six projects for "Mongolia" and "crowdsourcing," and one project for "Mongolia" and "online citizen science" (Table 1). Considering the overlap between search terms (Table 1), a total of 10 different projects were identified and used in the following qualitative analysis. The projects started between 1997 and 2019 (Table 1). The topics studied covered both the natural and social sciences. Projects focusing on natural aspects were mainly found under the keyword "citizen science” while projects focusing on social aspects were found under "crowdsourcing.” The similarities and differences may have been influenced by the similar, but not identical meanings of the keywords used [47]. The two projects, "Field Expedition Mongolia" and "The Mongolian Wordnet," are Mongolia-focused subprojects of international initiatives. Except for Public Lab Mongolia, which was initiated by OSM Mongolia and currently operates locally in Mongolia, the remaining seven projects are purely international.

**Table 1:** Projects identified through keyword search from 1991–2021.

Project	Keyword	Year mentioned in literatures (Number)	Year of project start	Geographic scope	Field	Research topic
CyberTracker	citizen science	2004(1), 2007(1), 2012(1), 2014(1)	1997	worldwide	Natural	animals
DustDuino	citizen science	2015(1)	2015	worldwide	Natural	air quality
	crowdsourcing	2015(1)				
Cyber-archaeology by CISA3 / Field Expedition Mongolia	citizen science	2011(2), 2012(2), 2013(5), 2015(2), 2016(2), 2017(3), 2019(2), 2020(3)	2007 / 2010	worldwide / Mongolia	Social	archaeology
	crowdsourcing	2011(2), 2012(2), 2013(14), 2014(7), 2015(7), 2016(5), 2017(3), 2018(3), 2019(2), 2020(2)				
	online citizen science	2015(1), 2016(1), 2017(1), 2019(1)				
Indicator Bats Program	citizen science	2013(1)	2005	worldwide	Natural	insects
InstantWILD	citizen science	2013(1)	2011	worldwide	Natural	animals
MyShake	citizen science	2016(1)	2016	worldwide	Natural	natural disasters
OSM Mongolia / Public Lab Mongolia	citizen science	2014(1), 2019(1), 2020(1)	2013 / 2018	Mongolia	Natural and Social	cities
	crowdsourcing	2014(1), 2019(1), 2020(1)				
TabaccoSpotter	crowdsourcing	2019(1)	2019	worldwide	Social	tabacco
Universal Knowledge Core / The Mongolian Wordnet	crowdsourcing	2015(1), 2018 (1)	2014 / 2016	worldwide / Mongolia	Social	language
What3words.com	crowdsourcing	2018(1)	2013	worldwide	Social	cities

#### Platform search

**Fig 4:**
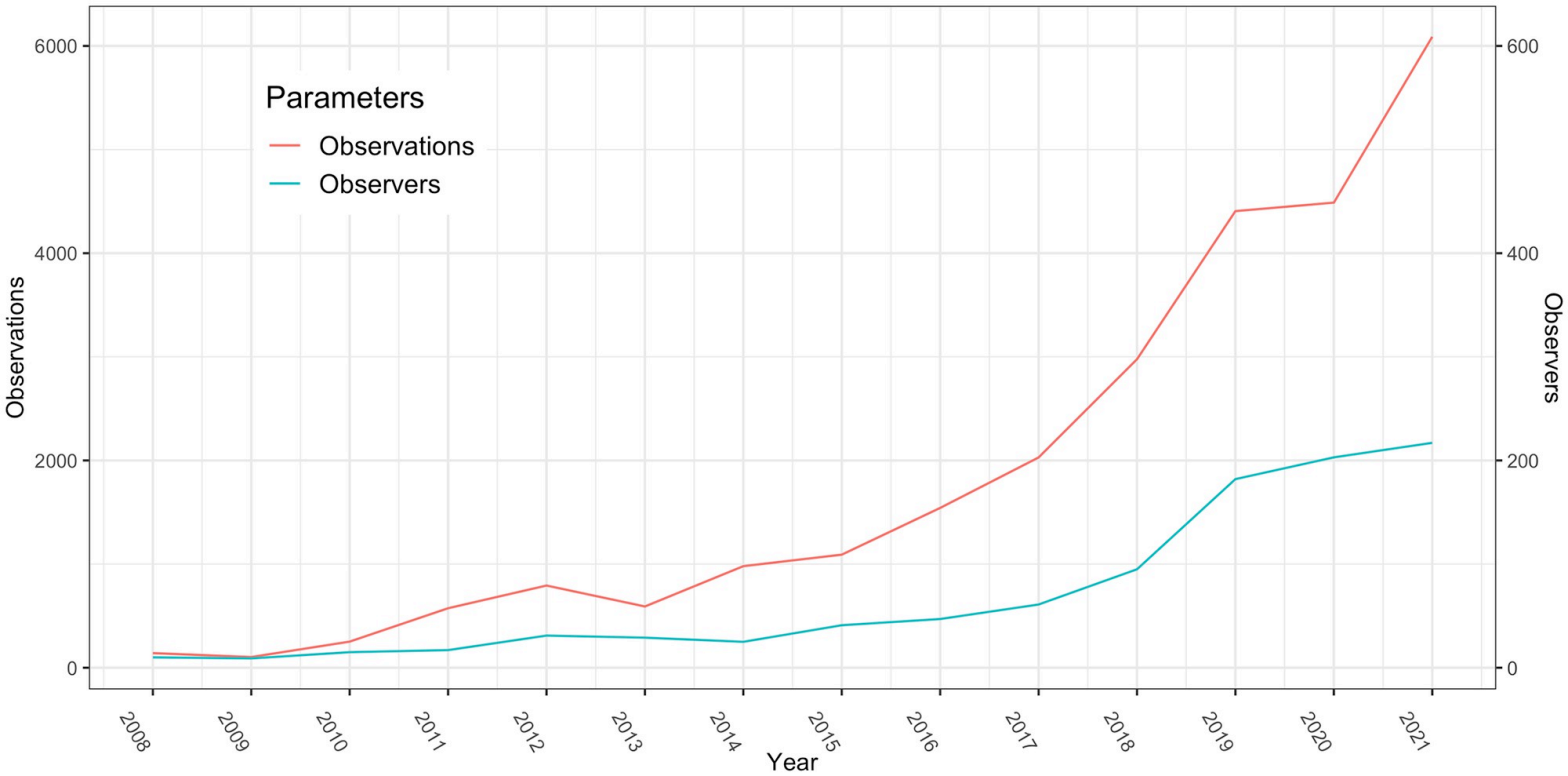
Time-series of the number of observations (left axis: Red line) and observers (right axis: Blue line) for Mongolia from iNaturalist from 2008–2021.

The number of observations and observers on iNaturalist has increased since 2010; this increase, especially in the last five years, is remarkable (Fig 4).

According to iNaturalist, approximately 700 observers are currently registered in Mongolia, with approximately 45,000 observations. Plants are by far the most commonly observed category (71%), followed by animals (14%), birds (6%), and insects (4%).

### Qualitative analysis

In terms of web technology, nine projects had websites, five had web applications, and five had mobile applications. Digital educational resources in the form of e-books and/or online encyclopedias/knowledge bases were found in only two projects (see Table 2). Locally operated Public Lab Mongolia offers in-person learning opportunities. Social media presence was implemented on Facebook in five projects, Twitter in six projects, Instagram in three projects, and LinkedIn in one project.

**Table 2:** Identified projects and their use of web technologies, link to UN SDGs, and Haklay’s classification.

Project	Use of web technologies			SDGs	Haklay’s classification
	web platforms	education	social media		
CyberTracker	website, mobile app	educational materials	Facebook	15, 9	2
DustDuino	website	N/A	N/A	3, 11, 9	2
Cyber-archaeology by CISA3 / Field Expedition Mongolia	website, web app	N/A	N/A	9	2
Indicator Bats Program	website	educational materials	Facebook, Twitter, Instagram	15	2
InstantWILD	web app, mobile app	N/A	Twitter	15, 9	2
MyShake	website, mobile app	N/A	Twitter	3, 11, 9	2
OSM Mongolia / Public Lab Mongolia	website, web spp	in-person learning	Facebook, Twitter, LinkedIn	11, 3	2, 3
TabaccoSpotter	mobile app	N/A	Facebook, Twitter, Instagram	3, 11	1
Universal Knowledge Core / The Mongolian Wordnet	web app	N/A	N/A	9	2
What3words.com	web app, mobile app	N/A	Facebook, Twitter, Instagram	11, 9	2

Regarding the UN SDGs, seven out of 10 projects can be assigned to SDG 9, “Industry, Innovation, and Infrastructure,” followed by four projects on SDG 3, “Projects in Health and Wellbeing,” four projects on SDG 11, “Sustainable Cities and Communities,” and three projects on SDG 15, “Life on Land” (Table 2).

According to Haklay’s classification, nine out of 10 projects can be classified as Level 2, “Distributed intelligence,” while only one project was classified as Level 1, “Crowdsourcing.” Public Lab Mongolia provides multiple participation opportunities; one of them can be attributed to Level 2, “Distributed intelligence,” and the other to Level 3, “Participatory science” (Table 2).

### Literature-based analysis

Citizen science in Germany has become well established over the last two decades [64]. Among the EU member states, Germany has the most advanced citizen science policies [36]. Citizen science in Japan is well-known for its disaster management projects, particularly the 2011 Safecast Project [38, 39]. NGOs, universities, and non-university research institutions are the main actors in citizen science in Germany [65] and Japan [66].

The natural sciences are thematically the most strongly represented in Germany, with the most frequently studied areas being biodiversity and environmental monitoring. Approximately 2,210,600 observations and 47,100 observers currently exist on iNatualist, with animals being the most commonly observed category (37%), followed by insects (22%), plants (22%), and birds (9%). Social sciences are represented to a slightly lesser extent by projects in the fields of history, humanities, and health [13, 67]. A steep increase in the number of observers and observations occurred in 2017.

In Japan, the most frequently studied field is natural sciences, with a focus on ecology [68]. In iNatualist, there are approximately 333,000 observations and 10,200 observers, with animals being the most commonly observed category (41%), followed by insects (22%), plants (18%), and birds (10%). Similar to Germany, the number of observers and observations has increased sharply since 2017.

With regard to the use of web technologies, online platforms (websites, web apps, and mobile apps), and social media are already well established in Germany and Japan, but there is still room for improvement in the further development of educational tools and the exchange of data between projects [20, 62].

According to Schleicher et al. (2020), 127 citizen science projects in Germany address 12 of the 17 SDGs. The two SDGs most frequently addressed by citizen science projects in Germany were SDG 15, “Life in Land” (48 projects), and SDG 4, “Good Education” (33 projects). In Japan, it is difficult to obtain a common idea of the SDGs in OCS projects. However, from other studies, a link between the SDGs and citizen science in Japan can be observed in frameworks such as Digital Earth [69] and Society 5.0 [70, 71], which focus on environmental and social aspects in the context of digitalization.

In terms of Haklay’s classification, the majority of projects in Germany and Japan fall into Level 2, "Distributed intelligence," and a minority fall into Level 1, "Crowdsourcing," and Level 3, "Participatory science” [63].

In contrast to Japan, Germany has a central platform, on which a high proportion of active projects (> 100) are presented [62]. This platform "Bürger schaffen Wissen" has been in operation since 2013 [37, 72], within which the state of citizen science and further potential in Germany has been examined from various angles [62–65].

## Discussion

This study focused on three aspects. First, the state of OCS in Mongolia was analyzed; second, it was placed in an international context compared with other countries. Based on the results, the potential of OCS to address environmental challenges in Mongolia is discussed.

A significant increase in the number of hits on Google Scholar and iNaturalist, as well as in the number of projects, has occurred in the last decade, indicating that citizen science in Mongolia is still relatively new. UN SDG 9, "Industry, Innovation, and Infrastructure," to which OCS projects are most frequently linked, reflects the general interest in innovative approaches to solve social and environmental challenges using new technologies [35].

When comparing Mongolia, Germany, and Japan, citizen science projects in all three countries focus on the natural sciences. Reviewing the iNaturalist platform, Mongolia has significantly fewer observations than Japan and Germany; however, it should be noted that the population in Mongolia is significantly smaller than that in other countries. A sharp increase in observers has occurred in all three countries since 2017. While there was a strong thematic focus on plants in Mongolia, there was a more balanced proportion of animal observations in Germany and Japan. Regarding the use of web technologies, projects in Mongolia follow general trends from other countries, such as Germany and Japan, which also apply to social media [20, 62]. Compared to Germany, projects in Mongolia address fewer UN SDGs; however, there are significantly fewer citizen science projects in Mongolia. In Haklay’s classification of the level of citizen participation and use of web technologies, all three countries are similar.

The progress of web technology is a driving factor in citizen science and crowdsourced research [73, 74]. Another factor is the overall positive international trend towards more citizen science [74, 75], which can be viewed as a driver of OCS activities in Mongolia. However, the proportions of international and local projects vary widely among countries. Compared to Mongolia, where the share of local projects is 10%, the share in Germany, for example, is significantly higher at approximately 70% [13].

The introduction of international projects can be a starting point of citizen science, whereas in the future, it is important to establish independent and local projects. In locally launched citizen science projects, the content covered can be better tailored to the interests and needs of local citizens. Ideally, data collected from local projects can be incorporated into international initiatives, such as the Global Biodiversity Information Facility (GBIF) database. Web technologies can facilitate local capacity building by opening new spaces for participation and making projects easier to establish owing to their cost-effectiveness and simplicity [76, 77]. A recent example of the emergence of local projects in the context of existing web-based platforms is the "Flora of Mongolia” project, which is part of iNaturalist [78].

Such local projects and citizen science can generally be a means of addressing environmental challenges, as they can raise citizen awareness of certain issues. For instance, citizen science can help people become aware of local issues related to climate change [13]. Increasing citizen understanding can help counter the effects of climate change by encouraging lifestyle adjustments. Citizens can complement measurement network obstacles by adding a large number of observations. Digital apps allow, for example, the supplementation of observations with images [79]. Extreme weather events have already occurred and will increase in the future, such as droughts and extreme cold; their impacts can be tracked locally and reported by citizens. This is particularly important in a sparsely populated country like Mongolia.

The effects of human activities, such as mining ecosystems, should be monitored regularly for sustainability. For example, citizens can participate in water source monitoring, including river and lake management, through photographic documentation [80]. The extent to which the composition of a species has changed can be observed in the context of citizen science in the form of applications that record biodiversity at various sites [81]. The successful use of citizen science for monitoring large-scale changes in land use and land cover was demonstrated in the LUCAS and FotoQuest Go Europe apps, in which users collected data via mobile devices and received financial compensation [82]. Such monitoring programs can be used to evaluate the effects of changes in livestock farming in Mongolia.

Herders in traditional livestock farming have indigenous ecological knowledge of the extensive grasslands in Mongolia. Including them in monitoring programs for landscape changes, as part of citizen science projects, would be highly useful [83]. Concerning migration from rural areas and herder aging, citizen science can help the next generation learn traditional and indigenous knowledge by collecting this knowledge within the framework of an app and making it accessible [84].

To facilitate the local production of daily products, citizen science can help to better connect local producers based on ICT systems [85]. Monitoring programs can be created within the framework of citizen science to obtain a better understanding of the emissions from heating systems and transport vehicles [86]. Based on the points shown, citizen science in Mongolia clearly holds a variety of potentials in addressing environmental challenges.

Further work could focus on identifying what we can learn of Mongolia from existing approaches using citizen-collected data from other countries, such as the development of smart cities in China [87] and how new directions can establish citizen science programs in other developing countries [15].

## Conclusions and outlook

This study provides an overview of the state of OCS in Mongolia by examining web technologies. Various web technologies are available for these projects. Most OCS projects in Mongolia have an international background, making Mongolia part of the international trend towards citizen science.

However, there are deficiencies in the development of national and local OCS projects in Mongolia. In particular, locally operated projects can improve measurement networks and foster an understanding of various environmentally and socially relevant challenges. A central platform where citizen science projects can present themselves, as implemented in Germany, would improve the network of citizen science. With such a platform, existing projects can be supported by making general guidelines available online while new impulses for other local projects can be provided.

## Supporting information

S1 Data (XLSX)
